# Frontiers in neuro-rehabilitation: neural basis of cognitive contributions to movement across neurological populations

**DOI:** 10.3389/fneur.2025.1638254

**Published:** 2025-07-04

**Authors:** Roberto Di Palma, Luigi Falco, Armando Coccia, Federica Amitrano, Gaetano Pagano, Giovanni D’Addio

**Affiliations:** ^1^Movement Analysis and Robotics Lab (MARLab), Istituti Clinici Scientifici Maugeri IRCCS, Telese Terme, Italy; ^2^Movement Analysis and Robotics Lab (MARLab), Istituti Clinici Scientifici Maugeri IRCCS, Bari, Italy

**Keywords:** active predictive coding, frontoparietal network, neuro-rehabilitation, dual-task training, executive functions

## Abstract

Cognitive-motor integration is essential for adaptive human behavior, involving reciprocal interactions between cognition and motor actions mediated by dynamic neural networks. The Active Predictive Coding (APC) framework highlights the bidirectional coupling of sensory inputs and motor actions, while the frontoparietal network, particularly the dorsolateral prefrontal cortex (DLPFC), plays a pivotal role in cognitive-motor tasks under high cognitive demands. Disturbances in this process have been observed in a variety of neurological conditions, resulting in inefficient neural adaptations and in-creased cognitive load. Rehabilitation strategies that integrate dual-task training, robotic devices and virtual reality (VR) have been shown to enhance neuroplasticity and recovery. To improve outcomes, neuro-rehabilitation must shift toward an interdisciplinary, personalized model that leverages neuroscientific and technological advancements to enhance recovery and quality of life.

## 1 Introduction

The interaction between cognitive processes and motor actions is fundamental to human behavior, ranging from simple locomotion to complex multitasking. This dynamic interplay is supported by hierarchically organized, interconnected neural networks spanning sensory, motor, and cognitive domains, which enable efficient and adaptable integration (1, 2). Cognition and motor functions engage in a bidirectional relationship, where motor actions refine cognitive processes, and cognitive control modulates motor outputs in real time. Key cognitive functions—including attention, executive control, and working memory—are integral to movement planning, error correction, and adaptive behavior during both routine and novel tasks (3). Clinical populations such as individuals with stroke, Parkinson’s disease, and Alzheimer’s disease often experience impairments in these cognitive domains, which directly affect motor performance and limit rehabilitation outcomes. This underscores the limitation of rehabilitation programs that address motor deficits in isolation, neglecting essential cognitive mechanisms critical for optimal recovery (4).

Recent advances in understanding the neural substrates of cognitive-motor integration, particularly through predictive coding and frontoparietal network dynamics, have significantly influenced rehabilitation approaches. The frontoparietal cortex, characterized by flexible and adaptive coding, supports behavioral flexibility by dynamically recoding task-relevant information on a trial-by-trial basis, underpinning cognitive control and predicting performance (5, 6). Predictive coding theory further elucidates how the brain hierarchically generates predictions about incoming stimuli, with frontoparietal regions orchestrating higher-level, contextual predictions distinct from those in sensory cortices during complex cognitive tasks such as working memory and language comprehension (7–9). Dynamic connectivity within this network supports both top-down predictions and bottom-up integration and can be externally modulated—for example, through brain stimulation—to enhance cognitive performance under demanding conditions (9). Oscillatory activity, particularly in the alpha band, facilitates these predictive coding processes by reflecting the recursive exchange of predictions and errors across hierarchical brain areas (10). Variability in frontoparietal network organization and synchronization has significant implications for individual differences in cognition and psychopathology (11–13).

These mechanistic insights have informed integrated cognitive-motor rehabilitation strategies. For instance, understanding how the dorsolateral prefrontal cortex (DLPFC) modulates motor output during cognitively demanding tasks has led to dual-task training paradigms, while insights from predictive coding have supported feedback-based approaches in virtual reality (VR) and robotics. Targeting both physical movement and the neural circuits underlying cognitive-motor integration holds promise for enhancing neuroplasticity and functional recovery in both healthy and clinical populations. This growing evidence base highlights the importance of incorporating neuropsychological assessment and cognitive training into neuro-rehabilitation.

Building on existing clinical and theoretical frameworks, this review adopts a narrative approach aimed at synthesizing and critically examining current evidence on the neural basis of cognitive-motor integration across neurological populations. The selection of sources was guided by clinical relevance, theoretical contribution, and recentness of publication. A literature search was conducted using PubMed, Scopus, and Web of Science.

Keywords included: “cognitive-motor integration,” “predictive coding,” “frontoparietal network,” “dual-task training,” “neuro-rehabilitation,” and “executive function.” Studies published between 2019 and 2024 were prioritized to reflect the most up-to-date evidence, although seminal or particularly influential earlier works were also included. Additional references were identified through a snowballing strategy, reviewing bibliographies of key articles.

The inclusion criteria were: (i) studies involving human or animal models with implications for clinical neuro-rehabilitation, (ii) relevance to cognitive control of movement, and (iii) focus on one or more neurological conditions (e.g., stroke, Parkinson’s disease, cognitive decline). Articles were excluded if they lacked original data, focused exclusively on pharmacological treatment without behavioral/rehabilitative implications, or were not published in peer-reviewed sources. Given the narrative nature of the review, no formal quality assessment or risk-of-bias analysis was performed. Instead, emphasis was placed on studies offering mechanistic insights, translational relevance, or emerging perspectives on neural network reorganization. This method allowed for a flexible yet clinically grounded overview of the interplay between cognition and motor function, as well as its implications for future rehabilitation strategies.

## 2 Neural mechanisms for sensorimotor and executive integration

### 2.1 Predictive coding in sensory-motor integration

Active Predictive Coding (APC) posits the neocortex as a predictive engine integrating sensory inputs and motor actions to anticipate environmental states and action consequences (14, 15). Prediction errors—discrepancies between expected and actual sensory inputs—are iteratively minimized across hierarchical cortical levels, driving adaptive behavior (14). Each cortical area contains both a state-prediction network estimating hidden sensory states and an action-prediction network generating actions based on these estimates. APC reframes perception and action as bidirectionally coupled and central to cognition. Visual search tasks illustrate how prior knowledge guides eye movements to optimize sensory acquisition and reduce uncertainty (1, 16).

Neurophysiological studies in rodents support APC: optogenetic and calcium imaging reveal that up to 40% of neurons in primary visual cortex (V1) encode predictive signals about voluntary movements, with task performance dropping by 60% upon optogenetic inhibition, highlighting early sensory-motor integration (17). While these findings may have limited direct generalizability to humans due to species differences, complementary computational and empirical studies show that neurons in cortical layers 2/3 integrate motor inputs with sensory feedback with 92% prediction accuracy, while layer 5 neurons refine predictions via feedback, reducing errors by 70% during sensory-motor tasks (1). Additionally, a 15-ms reduction in neuronal response latency during sensory-motor integration emphasizes the efficiency of feedback loops (1).

Oscillatory alpha-band activity has been implicated in APC, with traveling waves reflecting the recursive exchange of predictions and errors essential for temporal dynamics in adaptive sensory-motor integration (10).

### 2.2 The frontoparietal network and cognitive-motor control

The frontoparietal network is crucial for cognitive-motor tasks, enabling dynamic, context-sensitive modulation of motor outputs according to cognitive demands (18). DLPFC orchestrates these processes especially under multitasking and cognitive-motor interference conditions. Functional near-infrared spectroscopy (fNIRS) studies show increased DLPFC activity during dual-task scenarios, accompanied by enhanced connectivity to the inferior parietal cortex, reflecting heightened sensory-motor integration under cognitive load (18). Granger causality analyses reveal a top-down hierarchical flow from DLPFC to parietal regions, underscoring the network’s active role in coordinating predictive and executive functions necessary for adaptive behavior (18).

Research indicates that the frontoparietal network flexibly adapts to changing task demands by dynamically recoding task-relevant information on a trial-by-trial basis. This flexible coding, less stable during task switches, predicts behavioral performance and highlights the network’s central role in cognitive control and behavioral flexibility (5, 6). Predictive coding theory further attributes frontoparietal regions with generating higher-level, contextual predictions beyond those in sensory cortices, especially during complex tasks like working memory and language comprehension (7, 8). The network’s dynamic connectivity supports both top-down predictions and bottom-up integration and can be externally modulated via brain stimulation to enhance cognitive performance under demanding conditions (8, 9). Synchronization and oscillatory alpha-band activity are integral to these predictive coding mechanisms, facilitating recursive exchanges of predictions and prediction errors (10). Individual variability in frontoparietal network organization and synchronization significantly informs differences in cognition and susceptibility to psychopathology, with implications for personalized rehabilitation (11–13).

## 3 Implications for neurological populations

### 3.1 Parkinson’s disease: cognitive-motor dysregulation

Parkinson’s disease (PD) serves as a valuable clinical model for investigating disruptions in cognitive-motor integration (19). The dopaminergic degeneration that is characteristic of PD has been shown to affect not only the basal ganglia but also the cortical circuits involved in the planning and execution of motor actions (20). As demonstrated by Wang et al. (21), patients diagnosed with PD exhibit a 25% increase in functional connectivity between the prefrontal cortex (PFC) and other cortical areas during dual-task walking, compared to healthy controls (*p* < 0.01). This heightened reliance on the PFC suggests that cognitive resources are increasingly recruited to compensate for motor deficits, indicating a shift from automatic motor control to more conscious, effortful processing (21). Such a shift has been shown to impose cognitive strain, particularly during complex tasks, as evidenced by the inverse correlation between PFC connectivity and walking speed (*r* = −0.52, *p* < 0.01).

PD impairs both motor planning and execution, worsening gait function. For instance, Wang et al. (21) reported a 30% reduction in stride length and a 20% decrease in walking speed among PD patients compared to healthy controls (*p* < 0.001). Furthermore, the gain of the soleus H-reflex—a critical spinal reflex for locomotion—is reduced under dual-task conditions. In a separate study, Al-Yahya et al. (22) found that this reduction was 25% in healthy older adults (*p* < 0.05), but significantly more pronounced in PD patients, who exhibited a 40% decrease compared to their single-task baseline (*p* < 0.01). The present findings highlight the complex relationship between cortical compensation and peripheral motor deficits, emphasizing the importance of interventions that target both levels of control (22).

Beyond core motor symptoms, a range of non-motor manifestations also characterizes PD, including executive dysfunction, attentional deficits, and impaired working memory—all of which affect movement control and adaptability. Set-shifting difficulties and cognitive inflexibility, for example, are commonly associated with freezing of gait, particularly in novel or cognitively demanding environments (23).

Consistent with these observations, dual-task paradigms have consistently demonstrated that PD patients experience greater postural instability and reduced gait performance when simultaneously engaged in cognitive tasks, suggesting that frontostriatal dysfunction contributes to both motor and cognitive deficits (24).

These converging findings support the integration of cognitive training into rehabilitation strategies alongside motor exercises. Techniques such as external cueing, task-switching protocols, and dual-task training may enhance outcomes by improving cognitive-motor coordination. Recent evidence highlights the role of cognitive reserve (CR)—a construct shaped by education, occupational complexity, and intellectual engagement—as a key modulator of symptom severity and rehabilitation outcomes in PD (25–27). Higher CR is associated with preserved executive function, reduced motor impairment, and greater responsiveness to cognitively demanding interventions such as virtual reality and tele-rehabilitation (25, 28, 29). Conversely, individuals with lower CR may benefit more from conventional approaches, underscoring the need to tailor rehabilitation to individual cognitive profiles (30). Given its multidimensional nature and buffering capacity against both motor and non-motor symptoms (27, 31, 32), CR should be considered a critical factor in the design of personalized, cognitively informed therapeutic strategies (26, 29, 33).

### 3.2 Stroke: rewiring cognitive-motor pathways

Cognitive-motor integration is often impaired in stroke survivors due to focal damage in cortical or subcortical structures. A hallmark of post-stroke recovery is functional reorganization, whereby preserved cortical regions assume compensatory roles. A recent meta-analysis by Wang et al. (34), summarize that stroke patients exhibited a 40% increase in PFC activation during dual-task walking compared to healthy controls (*p* < 0.01), measured using fNIRS. This shift marks a compensatory adaptation in brain function, reflected in measurable changes in gait dynamics and motor efficiency.

While plasticity enables reorganization, compensatory pathways are often inefficient, reducing motor performance. The same meta-analysis reported a significant negative correlation between PFC activation and walking speed (*r* = −0.47, *p* < 0.05), highlighting the trade-off between increased cognitive load and reduced motor efficiency. Moreover, stroke survivors with severe motor-related cortical damage exhibited a 30% reduction in stride length compared to healthy individuals (*p* < 0.001), further emphasizing the impact of elevated cognitive demands on motor task performance. These motor outcomes not only highlight the cost of compensatory cognitive control but also provide key targets for designing more effective rehabilitation strategies. Neural plasticity allows for the formation of compensatory circuits, but these pathways typically demand increased cognitive effort to support motor functions. This increased reliance on the PFC during motor tasks is indicative of an adaptive yet inefficient strategy, underscoring the necessity for interventions that optimize the balance between cognitive load and motor performance in stroke rehabilitation (34, 35).

Beyond motor impairments, stroke frequently results in cognitive deficits, particularly in domains such as attention, executive function, and memory—all of which play a pivotal role in motor recovery and rehabilitation outcomes.

Executive and attentional deficits disrupt motor learning and adaptation. For instance, stroke survivors with limited working memory capacity may struggle to consolidate and retain motor strategies over time (36). Functional neuroimaging studies have revealed that successful motor relearning is associated with increased activation in both the prefrontal and parietal cortices, suggesting that these regions may support compensatory cognitive processes.

Dual-task paradigms—commonly used to assess cognitive-motor interference—frequently demonstrate that stroke patients exhibit reduced gait stability and walking speed when performing a concurrent cognitive task. This supports the notion that compromised cognitive resources directly affect motor execution (37).

Therefore, tailoring rehabilitation interventions to individual cognitive profiles—particularly by incorporating training for attention and executive function—may enhance motor recovery. Recent biomarker-based models support this integrative approach, aiming to align neuroplastic mechanisms with personalized therapeutic strategies (38).

### 3.3 Aging and cognitive decline: a diminishing reserve

In aging populations, the neural basis of cognitive-motor integration undergoes significant changes, including a 25% reduction in connectivity within the frontoparietal net-work compared to younger adults (*p* < 0.001) and a 30% increase in activity in regions associated with compensatory processes, such as the PFC (18). These alterations are particularly pronounced in neurodegenerative disorders like Alzheimer’s disease, where the capacity to perform dual tasks is significantly impaired due to compromised cortical control. Miura et al. (18) demonstrated that individuals with early-stage Alzheimer’s dis-ease exhibit a 40% reduction in primary motor cortex activity during dual-task walking (*p* < 0.01), accompanied by a 35% increase in PFC activation (*p* < 0.01), reflecting an over-reliance on compensatory mechanisms. Cognitive decline limits efficient resource allocation for dual tasks. Decreased basal ganglia and motor cortex activity further burdens compensatory areas such as the PFC, leading to slower walking speeds (a 25% reduction compared to controls, *p* < 0.001) and diminished task-switching efficiency. These findings underscore the maladaptive nature of compensatory neural activity in impaired motor-cognitive integration. Interventions that target enhancing neural plasticity and connectivity show promise in mitigating these deficits. Miura et al. (18) highlighted the potential of VR-based rehabilitation, which improved dual-task performance by 20% (*p* < 0.05) after an eight-week intervention in participants with mild cognitive impairment. The findings of this study lend support to the hypothesis that combining such strategies with physical exercise and cognitive training targeting multitasking capabilities may further facilitate the re-establishment of motor-cognitive integration in aging and neurodegenerative populations.

To synthesize the discussed evidence, Table 1 delineates the distinct neurophysiological and behavioral profiles associated with each condition, emphasizing how variations in prefrontal activation, gait dynamics, and frontoparietal connectivity reflect compensatory or maladaptive responses with direct clinical relevance.

**Table 1:** Summary of gait-related alterations and associated changes in prefrontal activation and frontoparietal connectivity as discussed in the preceding section.

Condition	PFC activation (↑/↓)	Gait speed	Step length	Frontoparietal connectivity	Clinical implications
Parkinson’s disease	↑ (+25%)	↓ (−20%)	↓ (−30%)	↑ (compensatory)	Increased reliance on conscious motor control due to reduced automaticity (21, 22); increased PFC activation during multitasking tasks (19, 20)
Stroke	↑ (+40%)	↓ (−15–30%)	↓ (−30%)	↑ (inefficient)	Inverse relationship between PFC activation and gait speed indicates reliance on less efficient neural circuits (34, 35)
Healthy aging	↑ (+30%)	↓ (−10–15%)	↔	↓ (−25%)	Decreased cognitive resources and neuroplasticity (18)
Early Alzheimer’s	↑ (+35%)	↓ (−25%)	↓	↓ (severely reduced)	Overburdened compensatory mechanisms (18)

↑ indicates an increase relative to healthy controls; ↓ indicates a decrease; ↔ denotes no significant change.

## 4 Discussion

Despite the well-established interconnection between cognitive and motor processes, rehabilitation practices remain largely compartmentalized. Physical therapy typically targets neuromotor restoration, while cognitive interventions address attention, executive function, and emotional regulation (39, 40). This divide remains despite evidence that cognitive deficits exacerbate motor impairments and delay recovery (41, 42).

Executive and attentional dysfunctions significantly compromise complex motor tasks and activities of daily living. Additionally, behavioral symptoms such as depression, apathy, and reduced cognitive flexibility can affect both patient adherence to therapy and long-term functional outcomes (39, 42).

These findings underscore the need for a unified rehabilitation model that treats cognitive and motor domains as dynamically interrelated, rather than isolated systems. Emerging technologies are beginning to challenge the limitations of this compartmentalized approach. Devices such as robotic exoskeletons, VR platforms, and wearable sensors facilitate the simultaneous engagement of cognitive and motor functions (43). These tools employ feedback-driven learning and task-specific adaptation to promote cortical reorganization (44, 45). VR, for instance, enhances gait while concurrently stimulating cognitive domains such as attention, memory, and visuospatial processing (46, 47). Likewise, robotic rehabilitation systems can deliver cognitive-motor dual-task training—where patients perform physical movements (e.g., walking or reaching) while engaging in concurrent cognitive challenges like arithmetic, memory recall, or decision-making (35, 48). Such engagement enhances neuroplasticity via activity-dependent sensorimotor and prefrontal mechanisms (49). These principles echo recent theoretical models suggesting that motor learning is not governed by isolated circuits, but rather by a coordinated interplay between the cerebellum, basal ganglia, and cortical areas. This ‘super-learning’ framework—grounded in the integration of supervised, reinforcement, and unsupervised learning—provides a neurophysiological foundation for designing multimodal rehabilitation approaches that target both cognitive and motor domains simultaneously (50).

Neuro-rehabilitation is thus evolving toward an integrated model that combines motor and cognitive therapies. Advanced technologies—such as robotic exoskeletons, dual-task protocols, and cognitive stimulation—offer promising, personalized treatment options, particularly for post-stroke populations. For example, Molteni et al. (51) demonstrated that subacute stroke patients using overground powered exoskeletons for 8 weeks (three sessions per week) achieved a 50% improvement in gait speed (*p* < 0.001) and a 40% increase in walking distance on the 6-min walk test (*p* < 0.01), compared to conventional therapy. These gains were paralleled by a 30% reduction in dual-task costs on gait speed (*p* < 0.05), indicating enhanced cognitive-motor integration (51).

Further evidence suggests that integrated approaches promote superior functional outcomes. Cognitive-motor dual-task paradigms have shown benefits across both healthy and clinical populations, improving motor adaptability, attentional control, and cognitive reserve (38). Complementary techniques like rhythmic auditory stimulation (RAS), which combine motor timing with attentional entrainment, have been associated with improved gait stability and sensorimotor integration in stroke and PD (35, 52). Such approaches may be particularly beneficial for individuals with higher CR, who are more likely to benefit from cognitively enriched motor training protocols.

Nonetheless, significant barriers remain. High costs, the need for specialized personnel, and technological complexity continue to limit large-scale implementation, especially in low-resource settings. Integrating these tools into existing healthcare systems requires logistical and infrastructural adjustments. Thus, while the clinical potential of these technologies is clear, their widespread adoption hinges on cost-effectiveness, sustainability, and accessibility.

Moreover, cognitive-motor integration is inherently complex in clinical practice. Dual-task protocols must be individualized, taking into account patients’ cognitive capacity, motivation, fatigue thresholds, and environmental context. Effective implementation also requires robust interdisciplinary collaboration among physical therapists, neuropsychologists, occupational therapists, and engineers.

Standardized guidelines are essential to support clinical decisions and tailor interventions. Updated training for clinicians is equally essential. Clinicians need technical and theoretical expertise in neurocognitive principles, dual-task methods, human–technology interaction, and sensor data interpretation. Continuous professional development will be critical to ensure the safe, effective, and ethical use of these emerging tools in routine care.

By harnessing the synergistic potential of motor and cognitive rehabilitation and embracing technological innovation, the field is poised to move toward a unified, interdisciplinary model. Such a model holds great promise not only for accelerating recovery but also for promoting long-term functional independence and enhancing quality of life in individuals with neurological disorders.

### 4.1 Limitations

The present narrative review is subject to certain inherent limitations. Notably, the absence of systematic inclusion criteria and the lack of formal quality assessment may introduce selection bias in the sources consulted (53). While the narrative approach enables a flexible synthesis of emerging evidence across diverse domains, it inherently limits the replicability and generalizability of findings compared to systematic reviews or meta-analyses. Moreover, a substantial portion of the reviewed literature is derived from studies employing heterogeneous methodologies and involving diverse clinical populations. This variability may affect the consistency and robustness of the conclusions drawn, underscoring the need for future research employing standardized protocols and well-defined outcome measures.

## 5 Conclusion

Cognitive impairments are frequently observed across a wide spectrum of neurological conditions and can significantly hinder motor recovery. This review underscores the intricate interconnection between cognitive and motor processes, supported by shared neural circuits. Recognizing this relationship informs rehabilitation strategies that engage both cognitive and motor systems.

To this end, clinicians are encouraged to incorporate neuropsychological assessments into standard rehabilitation protocols, enabling the identification of patients who may benefit from cognitively informed motor interventions. For instance, individuals with executive dysfunction may require structured environments and external cueing to support movement execution, whereas those with attentional deficits might benefit from gradual task progression and reduced environmental distractions.

The evidence reviewed also supports a transdiagnostic perspective, suggesting that cognitive contributions to motor function represent a shared mechanism across various neurological disorders, including stroke, Parkinson’s disease, and multiple sclerosis. This approach advocates for flexible, principle-based interventions that can be tailored to individual cognitive profiles, moving beyond rigid, diagnosis-driven frameworks.

Implementing cognitively informed rehabilitation requires clear pathways and adaptable protocols. Interdisciplinary collaboration and continued research are essential to refine these approaches and to identify reliable, clinically meaningful outcome measures.

In parallel, the evolving nature of rehabilitation necessitates a transformation in clinical training programs. Health professionals must be equipped not only with conventional therapeutic skills, but also with digital literacy and a solid grounding in neurocognitive principles that inform recovery.

While current research highlights the relevance of predictive coding, frontoparietal network reorganization, and dual-task paradigms in motor rehabilitation, further investigation is warranted. In particular, longitudinal studies are needed to examine the dynamic, bidirectional relationship between cognitive functioning and motor recovery. These studies should also assess the efficacy of dual-task interventions and explore the potential of emerging technologies—such as VR and brain–computer interfaces—to enhance cognitive–motor integration.

By systematically addressing the clinical, organizational, and educational challenges outlined in this review, the field can move toward more comprehensive, personalized, and accessible models of rehabilitation. Such advances hold the potential to significantly improve long-term functional outcomes and overall quality of life for individuals living with neurological disorders.
